# New strontium-based coatings show activity against pathogenic bacteria in spine infection

**DOI:** 10.3389/fbioe.2024.1347811

**Published:** 2024-04-10

**Authors:** Daniele Ghezzi, Gabriela Graziani, Martina Cappelletti, Inna V. Fadeeva, Matteo Montesissa, Enrico Sassoni, Giorgia Borciani, Katia Barbaro, Marco Boi, Nicola Baldini, Julietta V. Rau

**Affiliations:** ^1^ University of Bologna, Department of Pharmacy and Biotechnology, Bologna, Italy; ^2^ IRCCS Istituto Ortopedico Rizzoli, Biomedical Science and Technologies and Nanobiotechnology Lab, Bologna, Italy; ^3^ AA Baikov Institute of Metallurgy and Materials Science, Russian Academy of Sciences, Moscow, Russia; ^4^ University of Bologna, Department of Biomedical and Neuromotor Sciences, Bologna, Italy; ^5^ University of Bologna, Department of Civil, Chemical, Environmental and Materials Engineering, Bologna, Italy; ^6^ Istituto Zooprofilattico, Rome, Italy; ^7^ Istituto di Struttura della Materia, Consiglio Nazionale delle Ricerche (ISM-CNR), Rome, Italy

**Keywords:** infection, bacterial adhesion, spine, orthopaedics, antibacterial coatings, strontium tricalcium phosphate

## Abstract

Infections of implants and prostheses represent relevant complications associated with the implantation of biomedical devices in spine surgery. Indeed, due to the length of the surgical procedures and the need to implant invasive devices, infections have high incidence, interfere with osseointegration, and are becoming increasingly difficult to threat with common therapies due to the acquisition of antibiotic resistance genes by pathogenic bacteria. The application of metal-substituted tricalcium phosphate coatings onto the biomedical devices is a promising strategy to simultaneously prevent bacterial infections and promote osseointegration/osseoinduction. Strontium-substituted tricalcium phosphate (Sr-TCP) is known to be an encouraging formulation with osseoinductive properties, but its antimicrobial potential is still unexplored. To this end, novel Sr-TCP coatings were manufactured by Ionized Jet Deposition technology and characterized for their physiochemical and morphological properties, cytotoxicity, and bioactivity against *Escherichia coli* ATCC 8739 and *Staphylococcus aureus* ATCC 6538P human pathogenic strains. The coatings are nanostructured, as they are composed by aggregates with diameters from 90 nm up to 1 μm, and their morphology depends significantly on the deposition time. The Sr-TCP coatings did not exhibit any cytotoxic effects on human cell lines and provided an inhibitory effect on the planktonic growth of *E. coli* and *S. aureus* strains after 8 h of incubation. Furthermore, bacterial adhesion (after 4 h of exposure) and biofilm formation (after 24 h of cell growth) were significantly reduced when the strains were cultured on Sr-TCP compared to tricalcium phosphate only coatings. On Sr-TCP coatings, *E. coli* and *S. aureus* cells lost their organization in a biofilm-like structure and showed morphological alterations due to the toxic effect of the metal. These results demonstrate the stability and anti-adhesion/antibiofilm properties of IJD-manufactured Sr-TCP coatings, which represent potential candidates for future applications to prevent prostheses infections and to promote osteointegration/osteoinduction.

## 1 Introduction

Infections of prostheses are due to the colonization of pathogenic microorganisms such as bacteria, viruses, or fungi on or near a surgical implant, leading to infection and inflammation in the surrounding tissue (Tande and Patel, 2014). Prostheses can include artificial joints, heart valves, pacemakers, and other implanted medical devices (Lakkas et al., 2021). While these devices can significantly improve the quality of life for patients, they are also prone to bacterial colonization/contamination that can cause infections, limiting the functionality and durability of the device in addition to further medical complications if left untreated (Maki et al., 2008). In severe cases, an infected prosthesis may need to be revised, causing additional surgery and recovery time (Sousa and Abreu, 2018). Therefore, it is crucial to take precautions to prevent and manage infections of prostheses to ensure optimal outcomes and a successful recovery (Gallo and Nieslanikova, 2020).

Microbial biofilms are communities of microorganisms that adhere to surfaces and form complex structures (Donlan, 2002). Biofilms have become a significant concern in the medical field due to their presence on biomedical devices and being responsible of most hospital acquired infections and of most prostheses’ failures. The adhesion of bacteria and biofilm formation on these devices hamper the efficacy of traditional cleaning methods and antimicrobial treatments (Khatoon et al., 2018). This is particularly relevant in spine surgery, where incidence rates are high (up to 20%), revision surgeries are particularly severe, and the need to avoid damage to the spinal cord reduces surgical options (Luca et al., 2021).

To prevent bacterial infections and biofilms formation, inorganic coatings have become a popular solution in the medical industry (Uneputty et al., 2022). These coatings act as a barrier to prevent the growth and spread of bacteria on surfaces and devices. The use of coatings in the prevention of bacterial infections is becoming increasingly important as the demand for safe and hygienic medical equipment and surfaces increases (Pietsch et al., 2020; Boi et al., 2022). Most of inorganic coatings are functionalized with metallic materials such as titanium dioxide, silver, copper, and zinc oxide, which have natural antimicrobial properties (Graziani et al., 2021; 2024; La Torre et al., 2021; Ghezzi et al., 2023a; 2023b). Metals have been used for centuries in various medical applications and are now gaining attention thanks to their pleiotropic activities as antimicrobial agents (Harrison et al., 2007; Turner, 2017; Pormohammad et al., 2023). The unique physical and chemical properties of metals make them effective against a wide range of bacteria, viruses, and fungi (Lemire et al., 2013). These properties include thermal conductivity, biocompatibility, and the ability to disrupt cellular processes by releasing metal ions (Prasad et al., 2017). The use of metals as antibacterial agents is particularly relevant as the growing threat of antibiotic resistance requires new strategies for controlling infections (Evans and Kavanagh, 2021).

Strontium is a chemical element that is widely used for various applications in different industries. Strontium has been used as co-metal in the functionalization of silver-modified titanium implant surfaces thanks to its ability to lower the cytotoxic effect of silver towards the host cells without altering the antimicrobial properties and to promote osseointegration and bone regeneration (Zhao et al., 2020; Alshammari et al., 2021a; Baheiraei et al., 2021). Recently, some indications on the antibacterial properties of this metal have been reported, indicating strontium as a promising alternative to traditional antibiotics (Alshammari et al., 2021a; 2021b).

For implant associated infections, a promising strategy is to coat implants with biomimetic calcium phosphate (CP) materials based on less resorbable hydroxyapatite (HA) or more resorbable tricalcium phosphate (TCP), containing substitution/dopant ion possessing antimicrobial properties, such as Ag, Zn, Cu, Mn, Fe (Ag-TCP (Graziani et al., 2021), Zn-HA (Prosolov et al., 2018a; 2018b), Zn-TCP (Fadeeva et al., 2021a), Cu-HA (Prosolov et al., 2020), Cu-TCP (Russo et al., 2020), Mn-TCP (Rau et al., 2019), Fe-TCP (Uskoković et al., 2019; Antoniac et al., 2020). A recent review (Fosca et al., 2023) analyzed and critically evaluated the available literature on ion doped coatings for biomedical implant applications.

Adoption of substitution/doping strategy of calcium phosphates it is possible to obtain both osseoinductive and antibacterial characteristics into the same device (Rau et al., 2017). Among these, tricalcium phosphates have proved to provide high osteoconductive and osteoinductive performances along with sustained metal ions release (Graziani et al., 2021; Fadeeva et al., 2022). Previous studies assessed the properties of strontium-doped hydroxyapatite whiskers bioceramic as safe bone substitute for the treatment of osteoporotic bone defects, promoting local bone regeneration and implant osseointegration to a level that strontium ranelate can achieve (Zhao et al., 2020).

In this work, the physicochemical, morphological, antibacterial, antibiofilm, and cytocompatibility properties of newly developed strontium-substituted tricalcium phosphate coatings were assessed on titanium-aluminum-vanadium (Ti6Al4V) alloys for application in spine prostheses. These alloys were functionalized by using the Ionized Jet Deposition (IJD), a plasma-plume assisted technology able to deposit the target on a substrate preserving the stoichiometry and providing both a nanostructured and high specific surface to assure a continual release of the antibacterial agent (Graziani et al., 2019; 2021; Pagnotta et al., 2020; Sartori et al., 2021; Ghezzi et al., 2023a).

## 2 Materials and methods

### 2.1 Materials

Strontium-substituted TCP with Sr content of 2.8 wt% was synthesized by precipitation from aqueous salt solutions followed by heat treatment at 900 °C, according to reaction:

$$2.9\text{Ca}{\left({\text{NO}}_{3}\right)}_{2}+0.1\text{Sr}{\left({\text{NO}}_{3}\right)}_{2}+2{\left({\text{NH}}_{4}\right)}_{2}{\text{HPO}}_{4}+2{\text{NH}}_{4}\text{OH}\to {\text{Ca}}_{2.9}{\text{Sr}}_{0.1}{\left({\text{PO}}_{4}\right)}_{2}+6{\text{NH}}_{4}{\text{NO}}_{3}+2H_{2}O$$

For the synthesis, calcium nitrate, strontium nitrate, di-substituted ammonium phosphate and a 25% aqueous ammonia solution (all chemical grade, Sigma-Aldrich) were used. 290 mL of a 0.5 M solution of calcium nitrate and 10 mL of a 0.5 M solution of strontium nitrate were placed in a reactor equipped with propeller stirrer, dropping funnel and pH meter. 200 mL of a 0.5 M solution of di-ammonium phosphate was added dropwise to the reaction mixture with stirring.

The acidity of the reaction mixture was maintained at 6.5–6.7. A 25% of aqueous ammonia solution was applied as alkaline agent. After adding all the reagents, the reaction mixture was stirred for 1 h, then filtered with Buchner funnel, washed with distilled water, and dried in an oven at 110 °C for 15 h. The resulting reaction product was crushed in an agate mortar and calcined in a muffle furnace at 900 °C for 1 h.

To obtain ceramic Sr-TCP samples, the powders were first pressed in a steel mold at a pressure of 100 kg/cm^2^, and then sintered in a furnace at 1,200 °C for 3 h. The so-prepared Sr-TCP targets for depositions were 1 cm in diameter and 5 mm of thickness.

For target characterization several techniques were used. The X-ray diffraction spectra of the samples were recorded on an “UltimaIV” Rigaku X-ray diffractometer (Japan) with a vertical goniometer and a high-speed semiconductor detector “D/teX”. The XRD measurements were carried out in CuKα radiation in the angle range 2θ—9°–100° with a step of 0.02°. Detector movement speed was 2°/min. Phase analysis of the samples was carried out using the ICDD database in the SIeve software package. Quantitative phase analysis results obtained by the Rietveld method using the JANA2006 software (JANA, Inc., universal City, TX, United States).

The microstructure of ceramics after sintering was studied by scanning electron microscopy using a Tescan Vega II scanning electron microscope (Tescan, Brno, Czech Republic) with an INCA Energy 300 energy-dispersive microanalysis system (Oxford Instruments, United Kingdom).

Medical grade titanium alloy (Grade 23 Ti6Al4V ELI alloy) cylinders (3 mm of thickness and 5 mm of diameter, Citieffe S.r.l., Bologna, Italy) and plates (5 mm of thickness, 20 × 20 mm of side, Citieffe S.r.l., Bologna, Italy) are used as substrates for the deposition. A surface roughness (Ra) of 5 μm is specifically selected for Ti6Al4V alloy disks, as representative of that of orthopaedic implants. The substrates are cleaned through ultrasonic cleaning for 15 min in isopropyl alcohol and ethanol solution, and they are dried using a flow of nitrogen (purity level = 99.999%) to avoid solvent residuals.

### 2.2 Sr-TCP coating fabrication

The Sr-TCP coatings were realized by Ionized Jet Deposition (IJD) (Noivion Srl, Rovereto (TN), Italy). IJD permits to deposit nanometric thin films starting from a solid target material (a metallic or ceramic material). The target is ablated and ionized by pulsed electron beam, and the ionized material is accelerated through plasma plume towards the substrates where it grows to form nanostructured films, as previously described (Graziani et al., 2019). To clean the target surface from impurities, the first 5 min of deposition are performed on a shutter. The deposition parameters are set up based on previous studies on calcium phosphates, and, in particular, on silver-doped TCP deposition (Graziani et al., 2021): target—substrate distance 80 mm, electron beam frequency 7 Hz, working voltage 18 kV. Deposition times of 10, 20, 30, and 60 min have been tested (c-10, c-20, c-30, and c-60), since 10 min permits to obtain a total substrate coverage and 60 min is the limit time before cracks formation in the coatings. However, a bacterial planktonic growth pre-screening has shown that coatings below 30 min of deposition do not cause antibacterial inhibition, so they were not further characterized.

### 2.3 Solubility of Sr-TCP ceramic in model liquid

The solubility of Sr-TCP in a saline solution was investigated by monitoring the concentration of Ca^2+^ and Sr^2+^ ions. The saline solution composition consisted of 0.9% NaCl in a TRIS buffer with a pH of 7.4. Sintered ceramic (1 g) was placed in a container with 50 mL of saline solution and incubated in a thermostat at a physiological temperature of 37 °C for up to 30 days. The cumulative release amount of Ca^2+^ and Sr^2+^ ions was measured using inductively coupled plasma optical emission spectroscopy (ICP-OES), specifically the 720-ES axial spectrometer (Agilent Technologies, NY, United States).

### 2.4 Coating characterization

The morphology of the coatings deposited on cylindrical titanium alloys, in term of nanostructure and surface texture, is analyzed by a Field Emission Gun Scanning Electron Microscopy (FEG-SEM, Tescan Mira3, CZ, working distance 10 mm, voltage of 5 kV). Prior to SEM investigation, samples were sputter-coated with graphite, to avoid interference with Energy Dispersive X-ray Spectrometry (EDS) analyses. In particular, FEG-SEM observation of the coatings at 200,00x has permitted to calculate the maximum (Dm), minimum (dm) and average (da) diameter of the globular aggregates that compose the Sr-TCP films. The diameter measurement was performed using the ImageJ software (National Institutes of Health, United States). In addition, the diameter distribution was analyzed, and the aggregates diameter were divided in four different groups: d < 100 nm, 100 nm ≤ d < 250 nm, 250 nm ≤ d < 500 nm and 500 nm ≤ d < 1,000 nm. The percentage of aggregates having diameters within each range was calculated.

To analyze the amount of strontium in the coating, Energy Dispersive X-ray Spectrometry system (EDS, Bruker probe coupled with a field emission gun scanning electron microscope Tescan Mira 3) was used. Then, FT-IR spectra (Perkin Elmer Spectrum 1) were acquired in ATR mode with resolution of 4 cm^−1^, 16 scans and data interval of 1 cm^−1^.

Coatings adhesion to the substrate was analyzed by micro-scratch test (Micro-Scratch Tester, CSM Instruments, Anton Paar S.r.l., Peseux, Switzerland, equipped with a conical Rockwell C stylus with spherical apex indenter tip, with 120° angle and a sphere radius of 100 µm). The test was performed following ISO 20502:2016. Five different measurements were acquired on two different coated titanium alloy plates (c-30) and averaged. To obtain the measurements, a distance of 0.5 mm was kept between two subsequent measures, to avoid the alteration in result due to previous test track. An increasing normal load (0.01–10 N) was applied through the tip with a loading rate of 10 N/min and an indenter traverse speed of 10 mm/min. Worn tracks are observed with a reflected-light microscope (magnification 20x and 50x) to study the failure modes of the films and associate them with the value of the relevant critical normal load (Lc) at which they occur. In this case, an initial area was selected where the coating is plastically deformed by the load, but not subjected to cracking (plastic strain). Then, three different Lc values and failure behaviors were observed: Lc1 is associated to formation of cracks in the coating, Lc2 corresponds to coatings delamination (or spallation, with evident detachment of coating fragments), and Lc3 indicates a complete penetration of the indenter tip in the coating and hence, corresponds to the failure load.

To access the stability of the coatings, we immersed them in PBS, for 4 h, 24 h, and 72 h. Samples (in duplicate) were immersed in 1 mL of PBS for each timepoint, then rinsed with deionized water to remove crystallized PBS, sputter-coated with graphite and observed using FEG-SEM.

### 2.5 Preparation of the bacterial cultures

Two reference pathogenic bacterial strains were tested in this study, i.e., the gram-negative *E. coli (Escherichia coli)* ATCC 8739 and the gram-positive *S. aureus (Staphylococcus aureus)* ATCC 6538P strains. All bacterial cultures were conducted using liquid LB as growth medium, to which 1.5% w/v agar was added to generate solid LB plates. The study was carried out by inoculating a single colony of each strain grown on agar plate for 24 h in 50-mL tubes with 5 mL of LB liquid medium. Cultures were grown overnight at 37 C under agitation at 150 rpm and then diluted to reach specific optical density values measured at 600 nm (OD_600_).

### 2.6 Evaluation of bacterial planktonic cell growth

The cultures grown overnight were diluted to reach a concentration of 10^6^ Colony Forming Units (CFU) mL^−1^. The control (TCP alone) and coated (Sr-TCP) samples were placed at the bottom of 48-wells microplates that were filled with 400 µL bacterial suspension and then incubated for 8 h at 37 °C under shaking conditions (130 rpm). Serial dilutions of the liquid medium were transferred onto LB agar plates. The plates were then incubated for 24 h at 37 C before enumerating the number of CFU mL^−1^ to assess the viable bacterial cells present in the suspension after the exposure to the control and coated samples. Each experiment was performed in triplicate.

### 2.7 Evaluation of bacterial biofilm formation

For the antibiofilm assays, the bacterial suspensions grown for 18 h were diluted to reach OD_600_ of 0.03. Sterile alloys coated with TCP (control) and with Sr-TCP were inserted into 1 mL of the diluted suspension in 24-wells microplates. The microplates were incubated for 24 h at 37°C under gentle shaking (50 rpm). The alloys were then removed from the cultures to quantify the bacterial biofilm formation through crystal violet staining (Graziani et al., 2021; Ghezzi et al., 2023a). TCP and Sr-TCP coated alloys were removed from the cultures and rinsed twice with saline solution to wash away the non-attached cells. The biofilms were first fixed with 99% ethanol (v/v) for 10 min and then immersed in a 0.2% (w/v) crystal violet solution for 10 min at room temperature. The excess of unbound crystal violet was removed by washing the alloys three times with sterile water. The bound dye was recovered from the alloys with 33% acetic acid (v/v). The amount of biofilm was measured at optical density of 595 nm. The background staining was corrected by subtracting the mean value for crystal violet bound to negative controls. Each experiment was performed in triplicate.

### 2.8 Evaluation of bacterial adhesion

For the anti-adhesion assays, the bacterial suspensions grown 18 h were diluted to reach OD_600_ of 0.2. Sterile alloys coated with TCP (control) and with Sr-TCP were inserted into 1 mL of the diluted suspension in 24-wells microplates. The microplates were incubated for 4 h at 37 C under gentle shaking (50 rpm). The alloys were then removed from the cultures to quantify the viable adhered bacterial cells. Briefly, TCP and Sr-TCP coated alloys were removed from the cultures and rinsed with saline solution (0.85% NaCl (w/v)) to wash away the non-attached cells. The alloys were placed into tubes with 1 mL saline solution and sonicated for 5 min to detach the adhered cells. Serial dilutions were performed and transferred onto LB agar plates. The plates were incubated for 24 h at 37 °C before enumerating the number of CFU mL^−1^ to assess the viable bacterial cells adhered on TCP and Sr-TCP titanium alloys. Each experiment was performed in triplicate. Additionally, the anti-adhesion properties of the coatings were also investigated through crystal violet assay described in paragraph 2.7.

### 2.9 Scanning electron microscopy of bacteria

Scanning Electron Microscopy (SEM) analyses were performed on titanium alloys coated with Sr-TCP and TCP alone (as control experiment) after 4 h and 24 h of bacterial incubation. Briefly, the titanium alloys were soaked in 1 mL of a bacterial suspension with OD_600_ = 0.03 in 24-multiwells plates and incubated at 37 °C for 4 and 24 h. The titanium alloys were washed twice in PBS 0.1 M, fixed in PBS 0.1 M pH 7.2 with glutaraldehyde 2.5% for 2 h, washed in sterile water for 10 min, and air-dried.

### 2.10 Cytotoxicity

Cytotoxicity of the coatings was measured on titanium alloys coated with Sr-TCP, TCP only, and on uncoated titanium alloys, previously sterilized under UV ray for 2 h, using bone marrow-derived mesenchymal stromal cells (BM-MSCs, ATCC PCS-500-012TM, Manassas, VA, United States). BM-MSCs were expanded in culture and maintained in alpha-MEM medium (Minimum Essential Medium Eagle, Sigma, Milan, Italy) supplemented with 10% fetal bovine serum (FBS, Sigma, Milan, Italy), 100 U mL^−1^ penicillin and 100 μg mL^−1^ streptomycin (Euroclone, Milan, Italy) at 37 °C, 5% CO_2_, and 95% humidity, with medium changed every 3 days. BM-MSCs were seeded with a density of 2 × 10^4^ per well in a 24-well plate. The following day the conditioned medium (CM) obtained from the materials immersed in alpha-MEM medium for 24 h was added. Cell metabolic activity was monitored at t0, i.e., before adding the CM, and at t1, i.e., 24 h after maintenance in CM. Briefly, to monitor cell metabolic activity, Alamar Blue assay was performed. The culture medium was removed, replaced with the Alamar Blue solution prepared as 10% v/v in fresh culture medium, then incubated for 3 h at 37 °C, 5% CO_2_, and 95% humidity. The fluorescence intensity resulted from the conversion of resazurin to resorufin was quantified by a microplate spectrophotometer (Infinite F200 PRO, TECAN, Mannedorf, Switzerland) at 535 nm excitation and 590 nm emission wavelengths. Data are expressed as percentage cell viability normalized to the uncoated titanium alloys and reported as mean ± standard error.

### 2.11 Data analysis

All the microbiological and cytotoxicity results are expressed as average ±standard deviation (n = 3). Statistical significance was determined through one-way ANOVA in GraphPad Prism. Differences were considered significant when *p*-value <0.05.

## 3 Results and discussion

### 3.1 Characterization of Sr-TCP targets and coatings

In this work, we evaluated the biological and structural properties of novel Sr-based coatings, manufactured for the first time by the Ionized Jet Deposition technology.

Scanning Electron Miscoscopy (SEM) images showed that β-TCP and Sr-TCP targets had comparable morphology (Supplementary Figure S1). However, an Sr-content of 0.77 ± 0.15 at% Sr was detected in Sr-TCP target by EDS, which was absent in β-TCP. Ca/P ratio, as assessed by EDS, was 1.08 for both targets. XRD data indicated that the sample was essentially composed by TCP, with a minor component of calcium pyrophosphate (<1 wt%). The cumulative release of Ca^2+^ and Sr^2+^ ions from Sr-TCP ceramic after soaking in the saline solution for 1, 3, and 30 days was measured by ICP-OES, and the obtained data are presented in Supplementary Table S1. As observed, the concentration of Ca^2+^ ions remained relatively stable, averaging 8.3 mg/L, while a rising trend was noted for Sr^2+^ ions. The average cumulative release of Ca^2+^ ions was below the levels of ionized calcium in the blood serum, which range from 0.088 to 0.104 g/L (Peacock, 2010). The result obtained for Ca^2+^ ions is comparable to our previous data on Cu-TCP ceramic soaked in a TRIS buffer solution (Fadeeva et al., 2021b). Data of ion release also showed a sustained release of strontium in saline medium for over 30 days.

**FIGURE 1:**
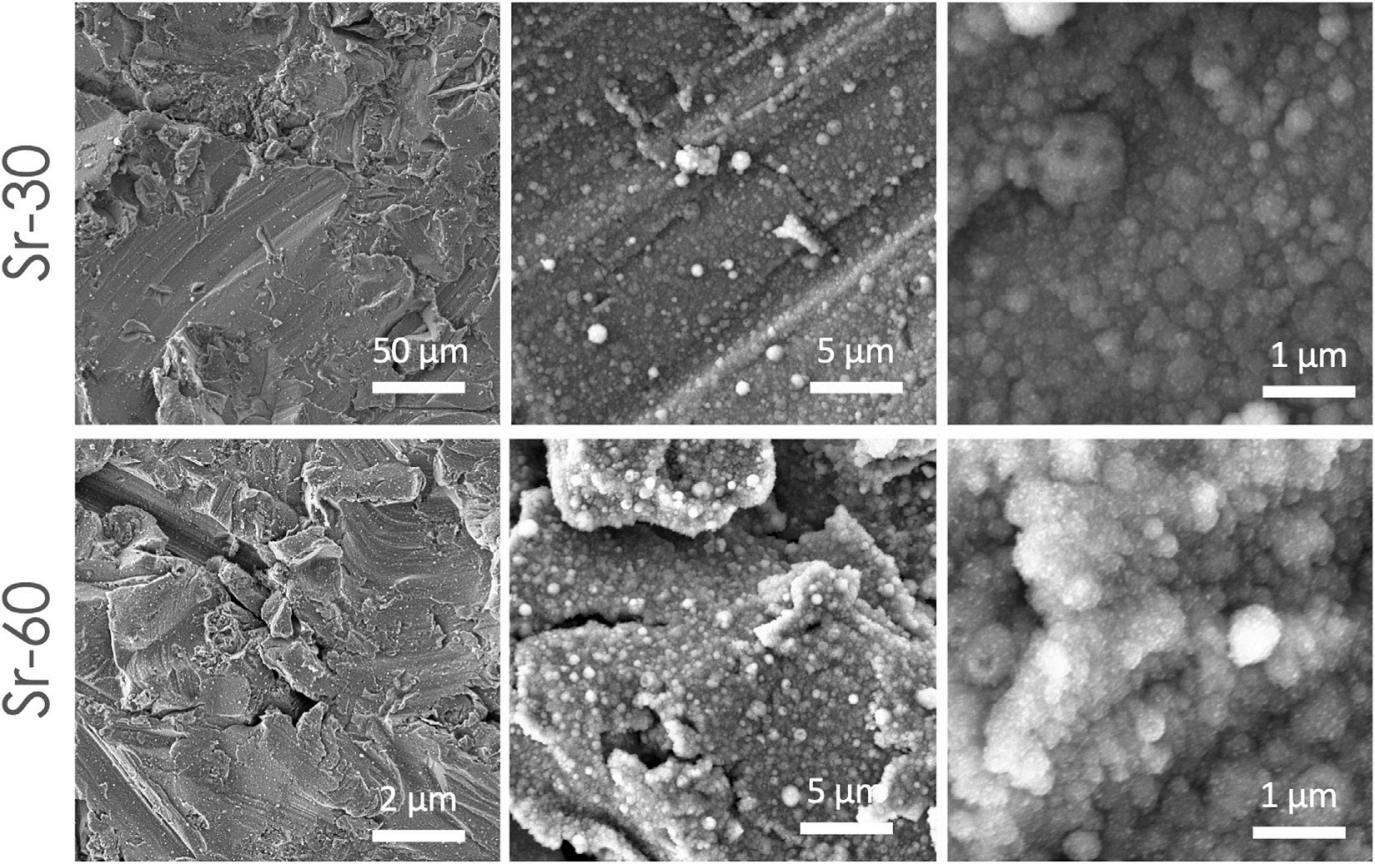
SEM images showing surface morphology of the coatings deposited at 30 and 60 min on titanium-aluminum-vanadium disks, at different magnification. SEM images were collected from samples surface without any preparation, except for metallization.

The results of FEG-SEM observations on the coatings are reported in Figure 1. All the coatings were composed by nanosized globular aggregates and showed high uniformity. Indeed, the substrate was covered by a homogeneous and uniform film formed by globular aggregates, and no uncovered parts nor surface defects (cracks or delamination) were observed for any of the deposition times. No significant differences were noticed among the different areas, i.e., in the centre and near the borders, at both deposition times (Supplementary Figure S2). The Sr-TCP coatings progressively dissolved upon immersion in the PBS medium but showed good stability over time, as demonstrated through SEM analyses in Supplementary Figure S3, in which coating is maintained after 4, 24, and 72 h of incubation with PBS.

In addition, the submicrometric thickness of the coatings permitted to preserve the original micro roughness finishing of the substrate surface and to obtain overall a multiscale (micro- and nano-) surface topography. At higher magnification it was possible to observe that the aggregates had a diameter in the 90 nm up to 1 µm range, with significant differences between coatings obtained at 30 and 60 min of deposition (Table 1). In fact, a lower deposition time resulted in lower aggregates diameter (minimum, maximum and average), indicating that the deposition time had an effect on the film nanostructure. This behaviour was noticed in other coatings manufactured by IJD setup (Graziani et al., 2021; Ghezzi et al., 2023a; Ghezzi et al., 2023b), where the amount of aggregates with finer diameter (d < 250 nm) decreased and grains with higher diameter (d > 500 nm) increased with deposition time.

**TABLE 1:** Aggregates diameter dimension and distribution.

	Dm	dm	da	Distribution (% of aggregates for each range)			
	(nm)	(nm)	(nm)	d < 100 nm	100 nm ≤ d < 250 nm	250 nm ≤ d < 500 nm	500 nm ≤ d < 1,000 nm
c-30	707 ± 242	95 ± 5	245 ± 117	2	64	30	4
c-60	960 ± 54	113 ± 13	284 ± 149	0	57	36	7

Comparing the morphology of Sr-TCP with that of Ag-TCP (both deposited at 30 min (Graziani et al., 2021)), we observed that the minimum, maximum, and average diameter are lower, indicating an effect of the specific composition and ion-doping on the coating morphology. At the same time, an effect is also noticed depending on the deposition target. Indeed, when targets are manufactured by compressed powders (Montesissa et al., 2023), instead of cements, a significant coarsening of the aggregates is observed, together with a higher inhomogeneity in aggregates dimensions.

EDS analyses showed the presence of calcium, phosphorous and strontium in the coatings, related to the deposition of the ion doped TCP. In particular, the amount of strontium in the films (∼0.150 wt%) (Table 2; Supplementary Figure S4) was the same at both deposition times, and lower compared to the nominal concentration in the starting target (∼2.8 wt%). FT-IR spectra (Supplementary Figure S5), show that the coating resembles the deposition target, so no decomposition phases are observed, but it has low crystallinity.

**TABLE 2:** EDS results of Sr-TCP target and coatings.

	Target	c-30	c-60
Sr (wt%)	2.80	0.145 ± 0.021	0.150 ± 0.014

Strontium was found in all areas of the target, with low variability among different zones. Longer deposition times resulted in lower variability, as already observed (Graziani et al., 2021). The percentage of strontium in the coatings was lower compared to the target. This behavior has already been observed for metal-doped TCP and was ascribed to different thermal conductivity between TCP and metals. Here, the reduction in Sr % (>50%) was higher than that found for other elements, such as silver, which suggested a preferential sputtering of calcium and phosphate over strontium and confirmed that the effect was element-dependent.

**FIGURE 2:**
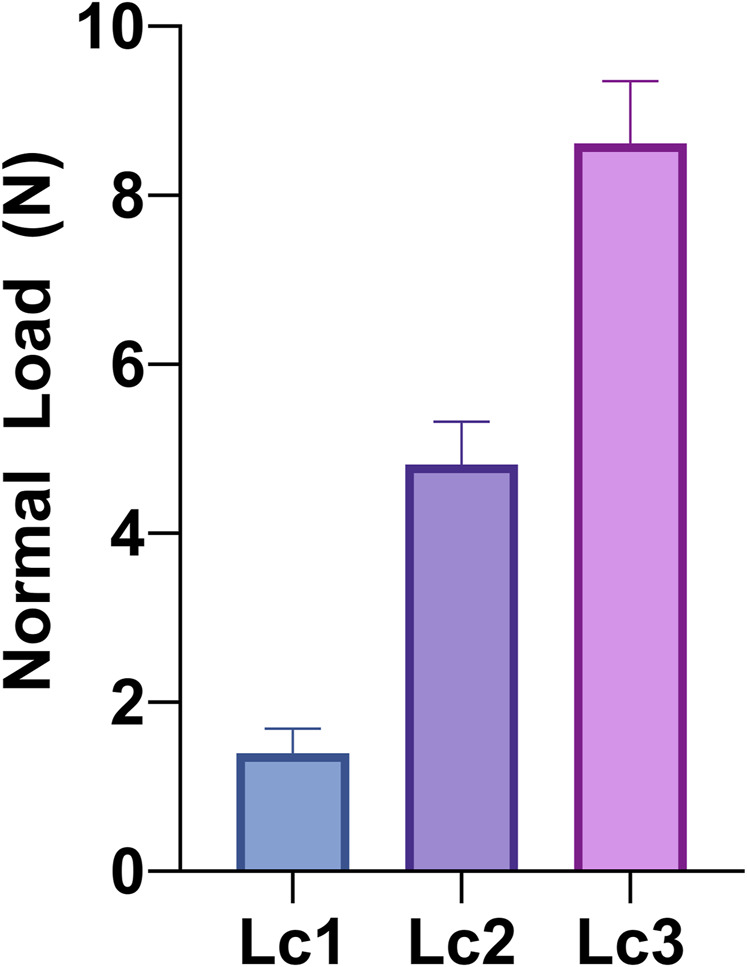
Critical normal loads (Lc) for Sr-TCP coating deposited for 30 min (c-30): Lc1 (formation of cracks in the coating), Lc2 (coatings delamination/spallation), and Lc3 (failure load). Values were measured by micro-scratch test following ISO 20502:2016 and averaging five different measurements from different coated titanium alloy plates.

The critical normal loads (Lc), recorded during the scratch test, are reported in Figure 2. We observed that, when the normal load was applied, the coating underwent an initial plastic deformation while remaining adhered to the substrate surface. As the normal force increased, cracks began to form in the coating (Lc1), then the coatings started delaminating (Lc2) up to the coating total detachment, which left the substrate surface bare (Lc3). Values of Lc1, Lc2, and Lc3 of 1.4 ± 0.3 N, 4.81 ± 0.5 N, and 8.6 ± 0.7 N are recorded (Figure 2). These values are in line with Literature references about HA coatings deposited by PLD (Fernández-Pradas et al., 2002; Johnson et al., 2006). As for the surface morphology, also in terms of adhesion we observed an increase compared to coatings obtained by deposition of pressed powders (Montesissa et al., 2023), further stressing the importance of the selection of the target. Indeed, in terms of adhesion, values found here for deposition at room temperature are in line with those reported after annealing (8.9 ± 0.8 N), in the case of pressed powders deposition.

### 3.2 Bioactivity analyses of Sr-TCP coatings

To study the effect of Sr-TCP on BM-MSCs, cell metabolic activity was assessed by Alamar Blue assay after 24 h from the cell seeding on the top of non-coated and coated alloys with TCP only (ctr-60) and Sr-TCP (c-60) at the highest deposition time investigated in this work, i.e., 60 min. Results are shown in Supplementary Figure S6, expressed as percentage of cell viability normalized on the non-coated alloy. Data of Alamar blue indicate that the conditioned medium of TCP and Sr-TCP does not impact on cells viability compared to non-coated alloys, indicating that the films were non-cytotoxic. In addition, no statistically significant reductions are observed in cell viability between the two coatings. This effect was likely associated with the bioactive composition of the coatings. Tricalcium phosphate promotes pro-osseoinductive activities, has higher solubility and dissolution rate compared to the most-used hydroxyapatite, and was proved to provide a more sustained metal release (Graziani et al., 2021). Therefore, the combined use of TCP with metals increased the coating biocompatibility. The cytocompatibility of Sr-based formulations was previously observed in the form of Sr-substituted tricalcium phosphate powder (Fadeeva et al., 2022) and Sr-substituted hydroxyapatite coating (De Bonis et al., 2020; Zhao et al., 2020). Sr^2+^ ions into the TCP released from the ceramics was shown to significantly improve human osteosarcoma MG-63 cell adhesion and proliferation after 24 h and 6 days of seeding, respectively (Fadeeva et al., 2022). Additional beneficial effects were demonstrated to be associated with strontium ions release, including the promotion of cell proliferation and angiogenesis and osteogenesis by increasing the secretion of cellular cytokines (Mao et al., 2017; Zhao et al., 2020; Borciani et al., 2022). Our coatings behave similarly as MSCs cells, in contact with the coatings show progressively increasing proliferation (Supplementary Figure S6). However, further cell studies will be carried out to assess eventual benefits of strontium in terms of cells differentiation towards an osteogenic lineage.

**FIGURE 3:**
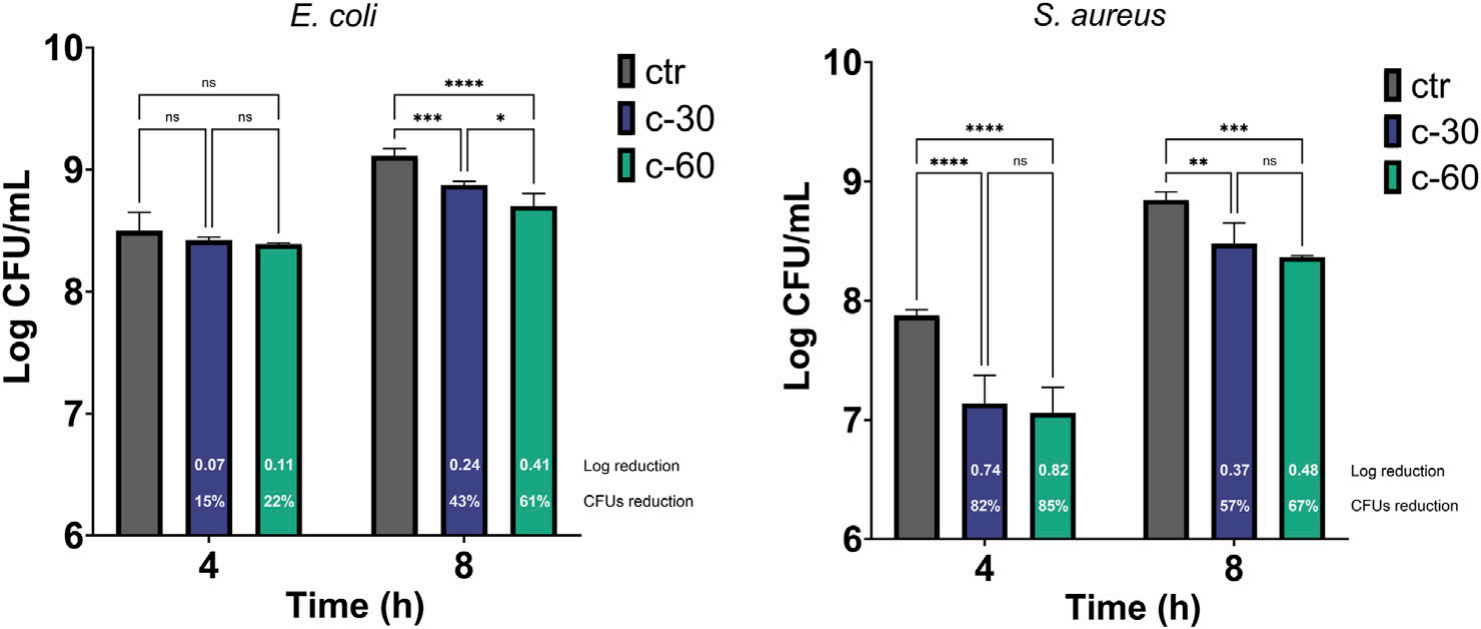
Antibacterial effect of Sr-TCP coated alloys on the bacterial planktonic growth. Two deposition times were tested, i.e., 30 min (c-30) and 60 min (c-60). Control samples (ctr) refer to titanium alloys coated with TCP at the highest deposition time tested (i.e., 60 min). The white numbers reported in the c-30 and c-60 columns refer to the logarithmic reduction values between the viable cells in the wells with Sr-TCP coating (c-30 and c-60) and those with TCP coatings (ctr-30 and ctr-60). Significant *p*-values are indicated with asterisks: ns (not significant) = *p* > 0.05; * = *p* < 0.05; ** = *p* < 0.01; *** = *p* < 0.001; **** = *p* < 0.0001.

Despite the recognized cytocompatibility, osteogenic, and angiogenic properties of strontium-substituted tricalcium phosphate, its antimicrobial potential has never been investigated so far. To this end, we performed antibacterial assays to evaluate any possible effect of Sr ions release in a liquid bacterial culture and the effect of the Sr-TCP coating on both adhesion and biofilm formation capacity of the bacterial cells. The antibacterial activity of Sr-TCP against the growth of *E. coli* ATCC 8739 and *S. aureus* ATCC 6538P was assessed after 4 h and 8 h of bacterial culture in the presence of titanium alloys coated with Sr-TCP deposited at 30 (c-30) and 60 min (c-60) or TCP (as control experiment) deposited at the highest deposition time (i.e., 60 min, “ctr” in Figure 3). The *E. coli* CFU count per mL was not significantly lower compared to the control in the presence of samples c-30 (0.07 logarithmic and 15% CFU reductions) and c-60 (0.11 log and 22% CFU reductions) at 4 h incubation, indicating that the amount of Sr^2+^ ions released in the medium did not inhibit the bacterial growth in the early timepoint. Distinctly, at 8 h incubation, the number of *E. coli* CFU per mL was significantly lower compared to the control in the presence of both c-30 (0.24 log and 43% CFU reductions) and c-60 (0.41 log and 61% CFU reductions). Conversely, the *S. aureus* growth was significantly inhibited at both the timepoints under analysis and with both Sr-TCP deposition times. After 4 h of exposure, the log reduction for *S. aureus* was 0.74 (i.e., 82% CFU reduction) in the presence of c-30 and 0.82 (85% CFU reduction) in the presence of c-60. After 8 h, the log reduction was 0.37 (57% CFU reduction) in the presence of c-30 and 0.48 (67% CFU reduction) in the presence of c-60 (Figure 3; Supplementary Table S2). These results indicate that, although we observed an increase in the planktonic growth of the cells of *E. coli* and *S. aureus* during the exposure to Sr-TCP, significant inhibition effects were detected compared to the control (TCP alone) by considering the cell count of each strain at each time point. Therefore, the newly developed Sr-TCP coatings seem to induce a delay in cell growth, but not a killing effect against planktonic cells. Similar with our results, previous studies investigating the antibacterial properties of strontium revealed a limited early inhibition of the bacterial growth (Alshammari et al., 2021a). The authors attributed this observation to a slow kinetic of release of Sr ions in the medium during bacterial growth. This previous finding could explain only slight inhibition effects we detected in this study after 4 h of exposure of *E. coli* and *S. aureus*.

**FIGURE 4:**
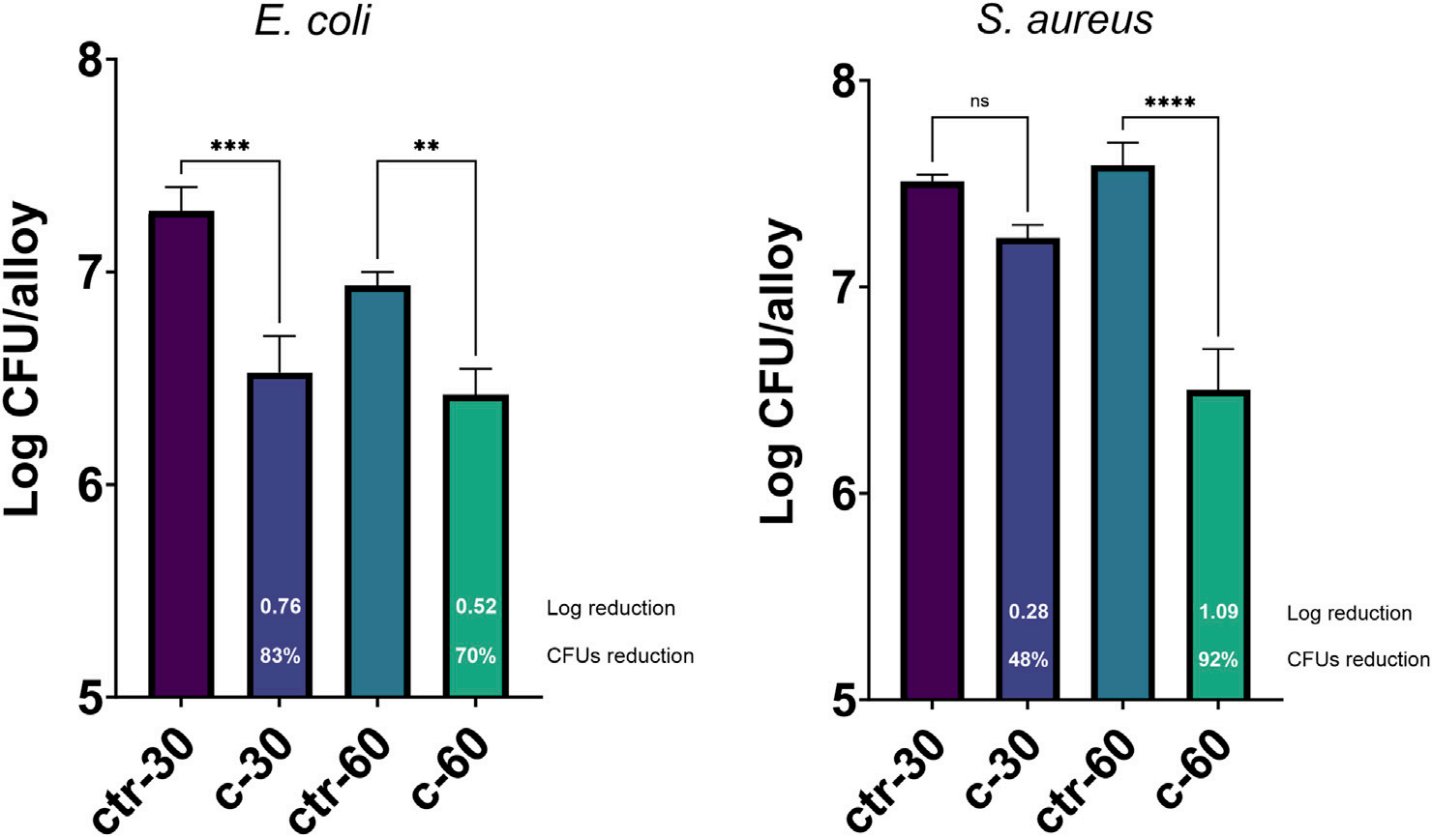
Bacterial cells adhesion assay on alloys coated with TCP and Sr-TCP. Two deposition times were tested for both TCP (ctr-30 and ctr-60) and Sr-TCP (c-30 and c-60) coatings. The white numbers reported in the c-30 and c-60 columns refer to the logarithmic reduction values between the viable cells on the Sr-TCP coatings (c-30 and c-60) and on the TCP coatings (ctr-30 and ctr-60). Significant *p*-values are indicated with asterisks: ns = *p* > 0.05; ** = *p* < 0.01; *** = *p* < 0.001; **** = *p* < 0.0001.

**FIGURE 5:**
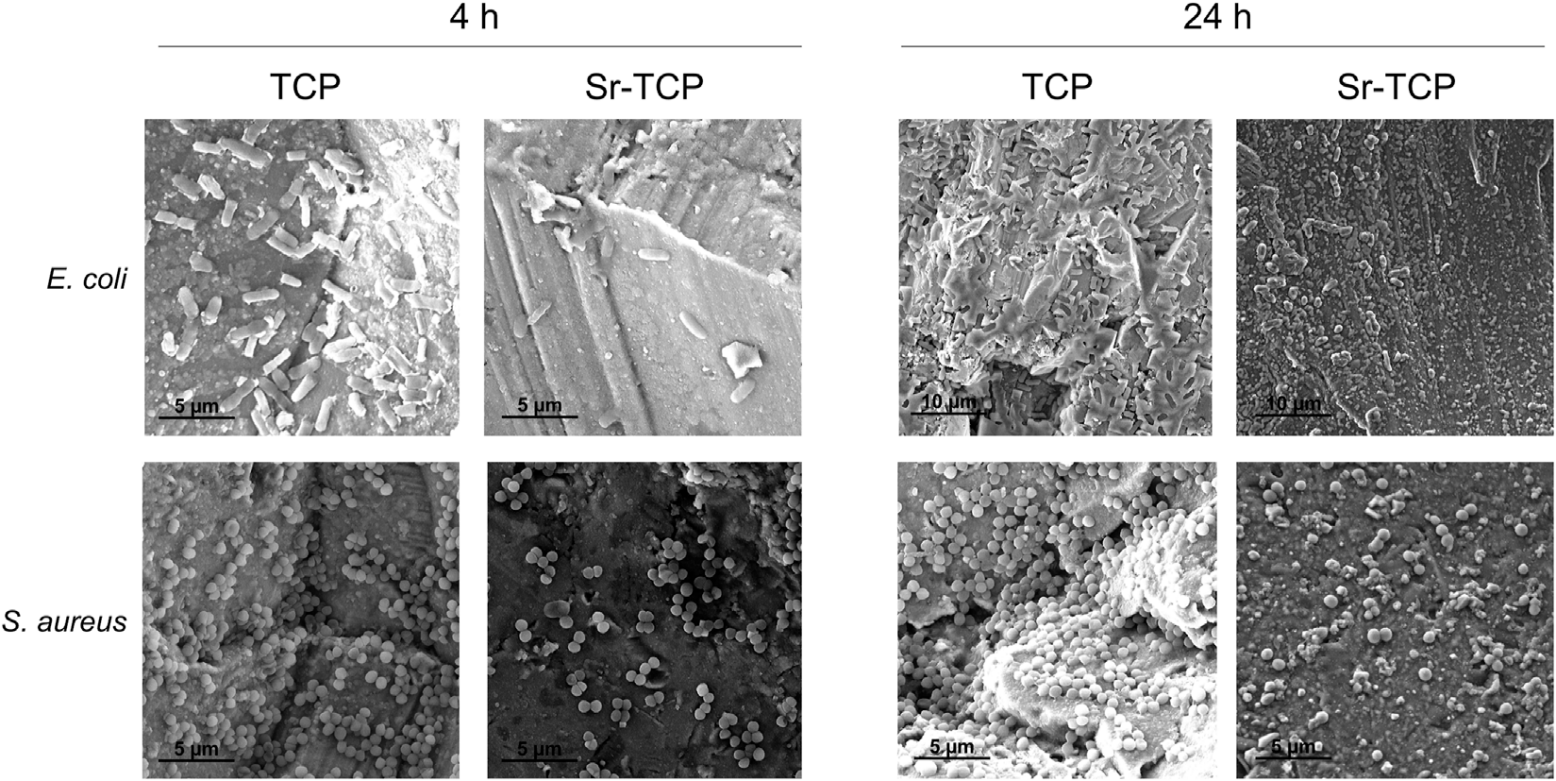
Microscopy images of *Escherichia coli* and *Staphylococcus aureus* cells after 4 h and 24 h of incubation on TCP and Sr-TCP coatings.

**FIGURE 6:**
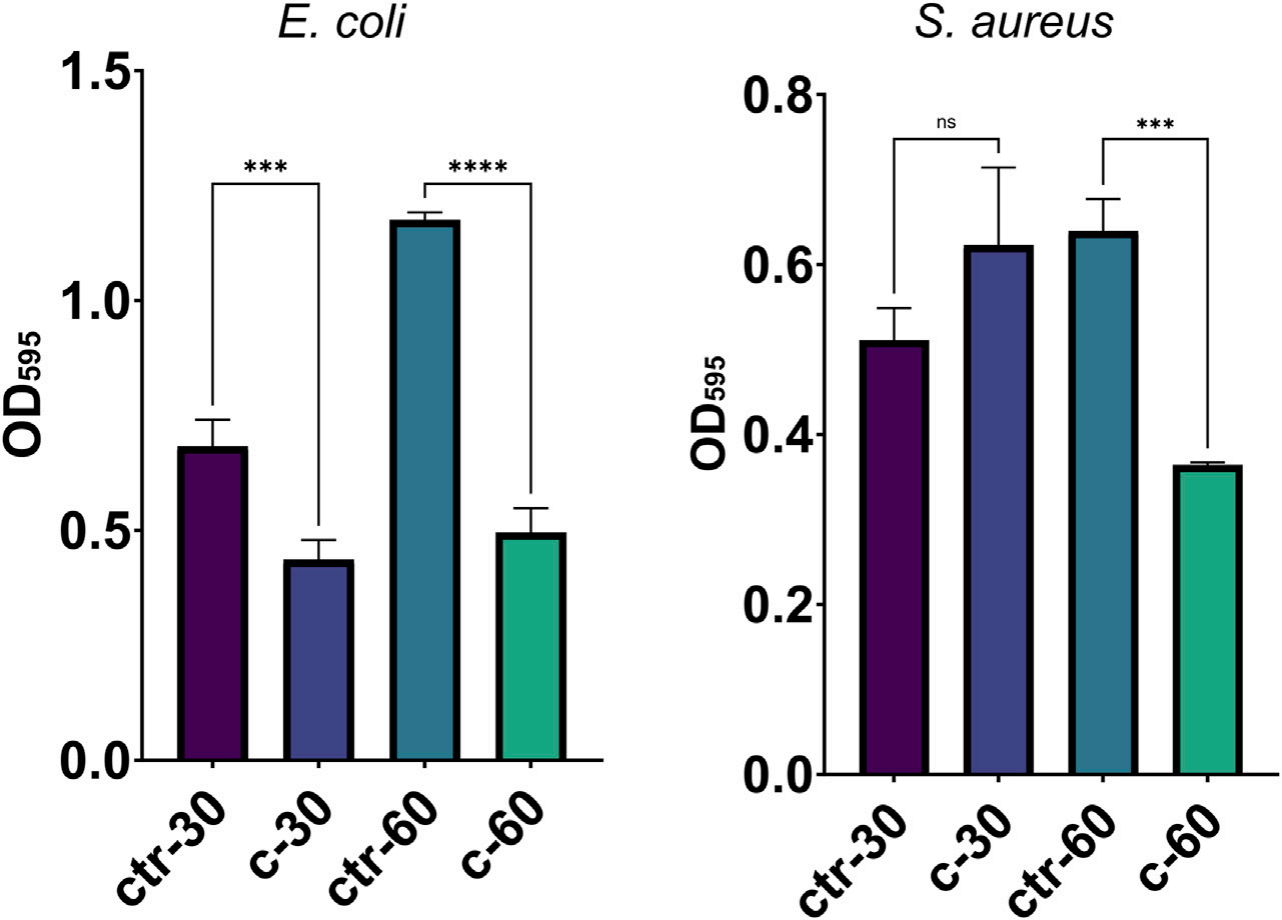
Bacterial biofilm formation on alloys coated with TCP and Sr-TCP. Two deposition times were tested for both TCP (ctr-30 and ctr-60) and Sr-TCP (c-30 and c-60) coatings. Significant *p*-values are indicated with asterisks: ns = *p* > 0.05; *** = *p* < 0.001; **** = *p* < 0.0001.

To assess the possible inhibition on the biofilm growth using Sr-TCP coatings as supports, we evaluated both the reduction of cells adhesion (after 4 h of incubation) and biofilm formation (after 24 h of incubation) on these supports compared to those on tricalcium phosphate (TCP) coatings without metal (as control). Our tests revealed that the reduction of *E. coli* viable adherent cells was significant on both c-30 (0.76 log reduction, 83% CFU reduction) and c-60 coatings (0.52 log reduction, 70% CFU reduction), with no significant differences between the two deposition times. In the case of *S. aureus* strain, the bacterial adhesion was reduced on c-30 (0.28 log reduction, 48% CFU reduction) compared to the control, although without statistical significance, whereas it significantly decreased on c-60 (1.09 log reduction, 92% CFU reduction) (Figure 4; Supplementary Table S3). In addition to the viable adherent bacterial cells count, we also performed a crystal violet (CV) staining of the biomass adhered on the coatings. CV-related data partly confirmed the bacterial reduction obtained from viable cell count, with c-60 providing the highest anti-adhesion activity against both *E. coli* (26% of reduced adhered biomass) and *S. aureus* (22% of reduction) (Supplementary Figure S7; Supplementary Table S3). Microscopy images also showed a lower number of bacteria attached to the Sr-TCP-coated surface compared to those attached to the TCP coating (Figure 5). Similar to the anti-adhesion observations, the antibiofilm activity of Sr-TCP coatings against *E. coli* was significant in the presence of Sr-TCP at both 30 and 60 min of deposition (36% and 58% of biofilm biomass reduction, respectively), while only c-60 had significant antibiofilm activity against *S. aureus* (43% of biofilm biomass reduction) (Figure 6; Table 3). Microscopy images also confirmed the anti-biofilm activity of Sr-TCP coatings. Indeed, a lower number of adherent cells were observed in the bioactive coating. Furthermore, the cells looked damaged and were not clustered in typical biofilm-like structures that are evident on TCP coatings (Figure 5).

**TABLE 3:** Antibiofilm activity of Sr-TCP coated alloys against bacterial cells.

Strain	Sample	% Reduced biofilm^a^	*p*-value^b^
*E. coli*	c-30	36.10	0.0007 (***)
	c-60	57.79	<0.0001 (****)
*S. aureus*	c-30	−21.92	0.1170 (ns)
	c-60	43.08	0.0010 (***)

^a^ The percentage of reduced biofilm refers to the ratio between the OD_595_ values of the CV-stained biofilm formed on the Sr-TCP, coating compared to the biofilm formed on the TCP, coating.

^b^ Significant *p*-values are indicated with asterisks: ns = *p* > 0.05; *** = *p* < 0.001; **** = *p* < 0.0001.

*E. coli* and *S. aureus* strains are known to cause severe orthopaedic infections also thanks to their ability to form biofilm both on the human tissues and on the materials applied for the prostheses (Tande and Patel, 2014). The reduction of bacterial adhesion is expected to have a significant impact on the reduction of biofilm formation *in vitro* and *in vivo*, as the adhesion represents the first stage of the biofilm formation process (Crouzet et al., 2014). We combined several tests targeting the inhibition of the planktonic growth with the inhibition of the adhesion and antibiofilm formation and we observed differences between the behavior of the two strains we tested, one gram-positive *S. aureus* strain and one gram-negative *E. coli* strain. Indeed, if on one hand the inhibition of the planktonic growth in the presence of Sr-TCP was generally higher in *S. aureus*, on the other hand the opposite scenario was observed in the anti-adhesion and antibiofilm assays, being *E. coli* cells less capable to adhere and form biofilm on the metal coating compared to *S. aureus* cells. In the presence of Sr-TCP coatings deposited for 30 min (c-30), *S. aureus* was less sensitive to the deposited metal in the adhesion assay compared to *E. coli*. This difference was previously observed in anti-adhesion studies performed on titanium alloys coated for 30 min of deposition with a different metal combined with TCP, i.e., silver-substituted tricalcium phosphate (Graziani et al., 2021), indicating a greater tolerance of *S. aureus* cells to action of direct contact with these metals in the initial stages of surface attachment. Additionally, we observed a higher amount of biofilm formed by *S. aureus* on c-30 Sr-TCP coatings compared to TCP alone, indicating that the contribution of the roughness in the biofilm formation process on c-30 is higher than the antibacterial activity of the metal. Conversely, *S. aureus* cells adhesion and biofilm formation were more hampered by the antibacterial metal than promoted by surface roughness in the presence of Sr-TCP coatings deposited for 60 min (c-60). These outcomes might be due to the different response of the two tested strains during the first hours of direct contact with the coated surface. Generally speaking, gram-positive strains, like those belonging to the *Staphylococcus* genus, possess teichoic acids in their cell wall that have a crucial role in cell adhesion onto abiotic surfaces in the first stages of the biofilm formation process (Fedtke et al., 2007; Sauer et al., 2022). These components might positively impact on the adhesion efficacy of *S. aureus* cells onto the Sr-TCP coatings, thus contributing to a lower sensitivity of *S. aureus* to the anti-adhesion and antibiofilm activities of the coating. Further explanations may be associated with the different response of the bacterial strains to the surface roughness of the Sr-TCP coating. Indeed, higher deposition times correspond to both higher amount of deposited metal, i.e., higher antimicrobial potential, and higher surface roughness, i.e., wider available surface for bacterial cells adhesion and biofilm formation (Yoda et al., 2014; Wu et al., 2018; Graziani et al., 2021). Additional effects of strontium on the bacterial cells can be described based on SEM images, in which we observed a low number of bacterial cells attached to the Sr-TCP coated surface compared to TCP after 4 h of exposure. Besides the lower number of adherent cells, after 24 h of incubation on Sr-TCP, cells of both *E. coli* and *S. aureus* were not organized in a biofilm-like structure, which, on the contrary, was visible on the TCP coated sample. Some bacterial cells on Sr-TCP coating also showed cracks and alterations in their rod-shape (*E. coli*) and spherical (*S. aureus*) morphology. Changes in the cell morphology may be either a consequence of the disruption and loss of functionality of the bacterial cells or an attempt of the bacterial cells to tolerate the toxicity of the metal present on the surface by modifying its cellular structure (Mathivanan et al., 2021; Pagnucco et al., 2023). Based on what is known for other toxic metals, strontium might also inhibit the expression of genes involved in bacterial cell wall formation and production of septal peptidoglycan during cell division with possible effects on cellular morphology (Ahmed et al., 2020).

## 4 Conclusion

Nanostructured thin films starting from Sr-TCP (2.8 wt%) targets were successfully deposited by the Ionized Jet Deposition technology onto Ti6Al4V alloys. Strontium is not generally recognized for its direct antibacterial properties. Instead, it is more commonly associated with other biological effects, particularly its osteoinductive properties, meaning it promotes bone growth and regeneration. We demonstrated that the Sr-TCP coatings developed and characterized in this study are not cytotoxic towards mesenchymal stem cells and possess potential osteoinductive activity. Additionally, the newly developed Sr-TCP coatings possess all the characteristics needed in terms of composition, surface morphology and roughness, absence of defects, submicrometric thickness, and high reproducibility, suitable for applications in spine surgery. We have also demonstrated that Sr-TCP coatings inhibit the growth of representative strains of *E. coli* and *S. aureus* species involved in prosthesis infections. Although the log reduction induced by the Sr coatings presented in this work is not sufficient for clinical use based on standard requirements (1 log reduction instead of >3 log reduction), these coatings represent good candidates for novel antimicrobial formulations including strontium together with other metals or antimicrobial compounds with boosted activity against bacterial adhesion. Indeed, these coatings exert significant anti-adhesive and antibiofilm properties by altering cell morphology and preventing the formation of biofilm-like structures as observed through microscopy analyses. Our future research will focus on determining the optimal amount of Sr^2+^ ions in synergy with additional dopant ions, such as Cu^2+^, Zn^2+^, and Ag^+^, to improve antibacterial activities as required by implant use.

## Data Availability

The original contributions presented in the study are included in the article/Supplementary Material, further inquiries can be directed to the corresponding authors.
