# Supplementary figures and images for: New strontium-based coatings show activity against pathogenic bacteria in spine infection
Source: Front Bioeng Biotechnol. 2024 Apr 10;12:1347811. doi: 10.3389/fbioe.2024.1347811 (PMC11044685; doi:10.3389/fbioe.2024.1347811)

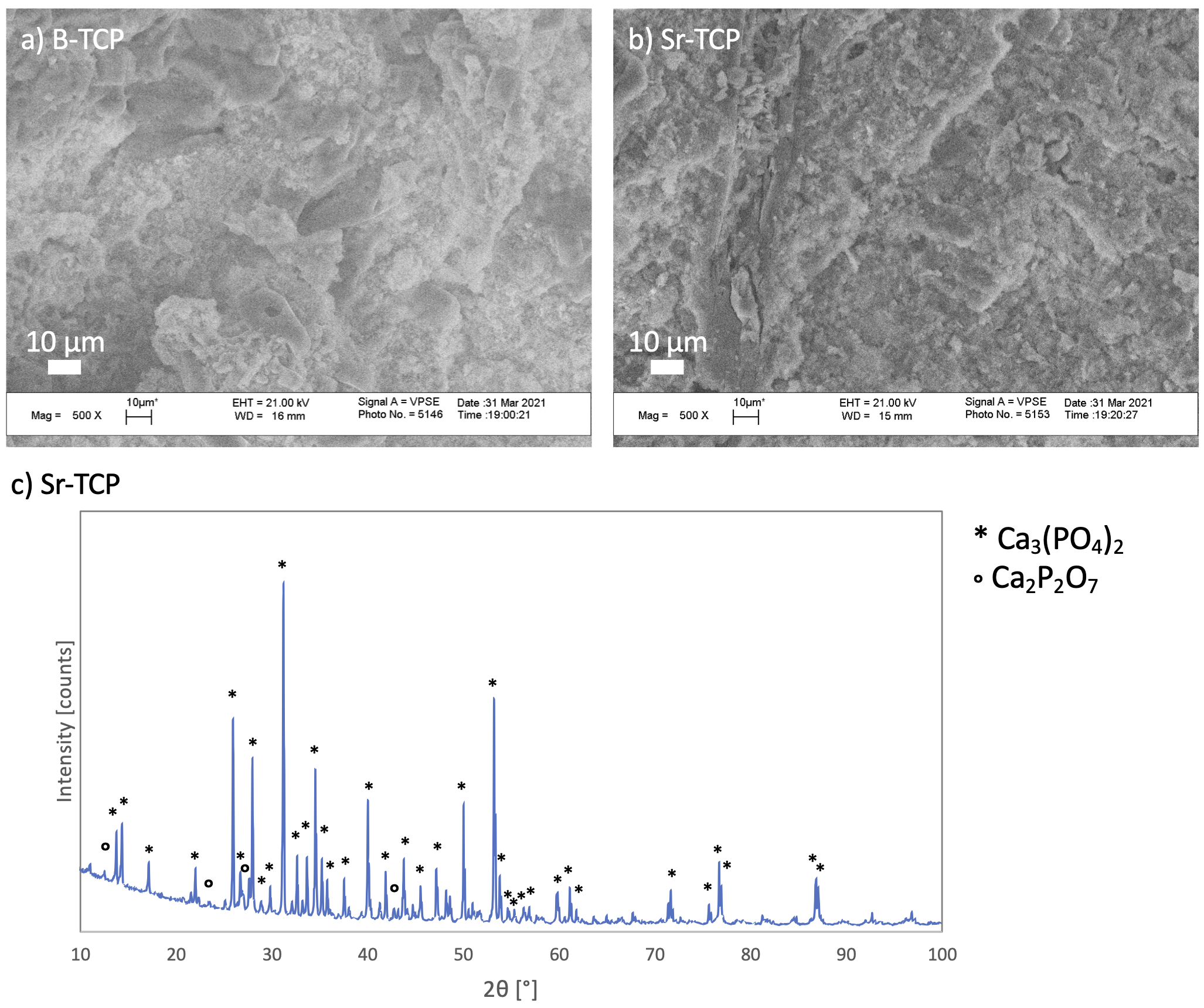

Supplement: Supplementary file 1 [file Image1.TIFF]

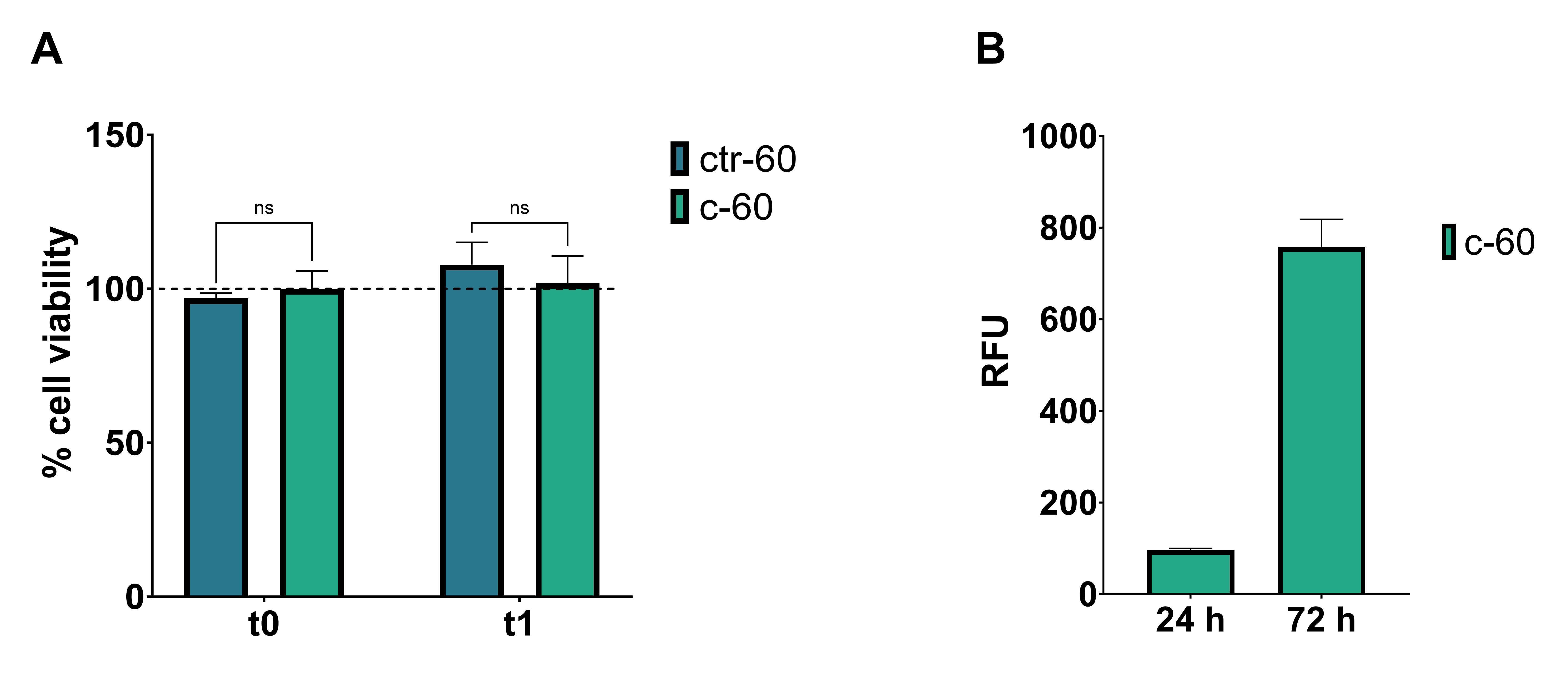

Supplement: Supplementary file 2 [file Image6.TIF]

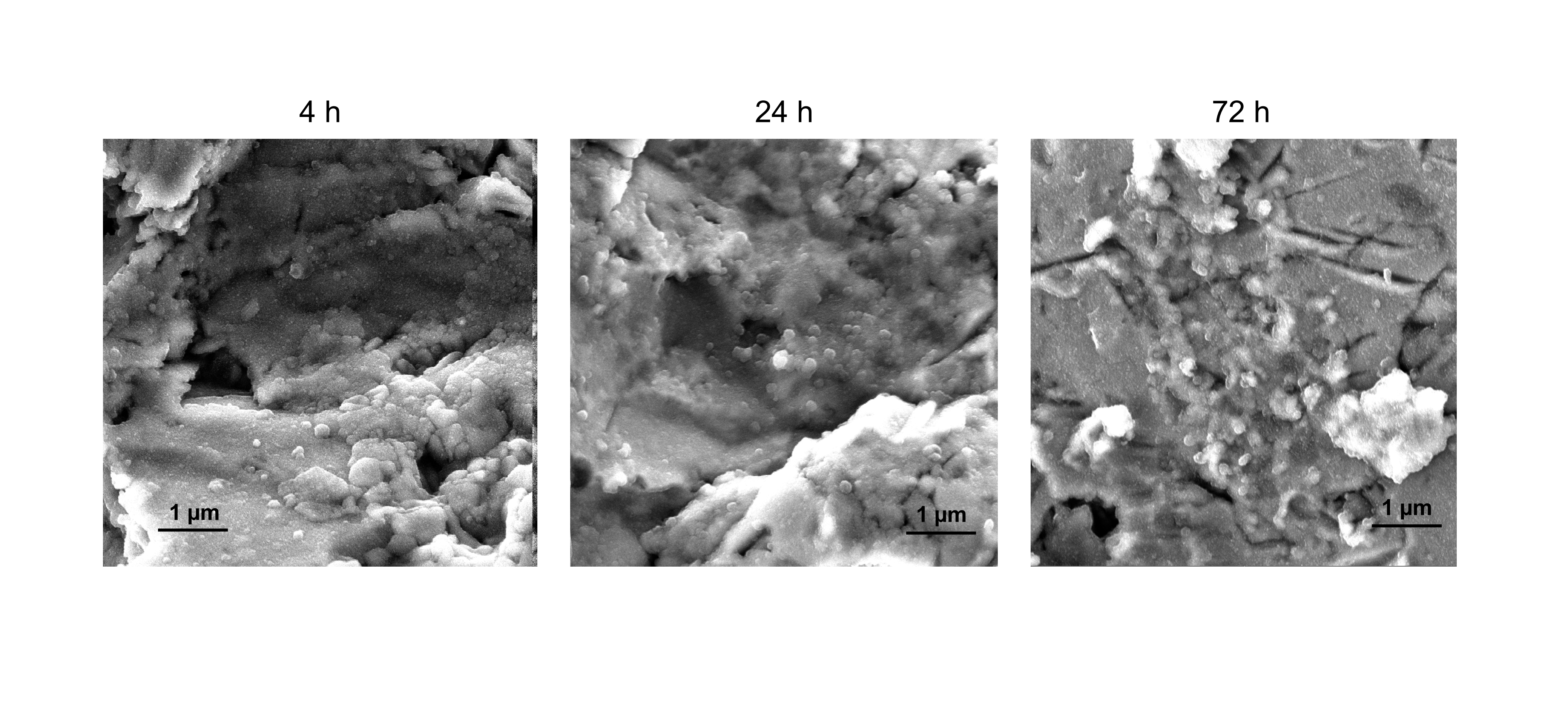

Supplement: Supplementary file 4 [file Image3.TIF]

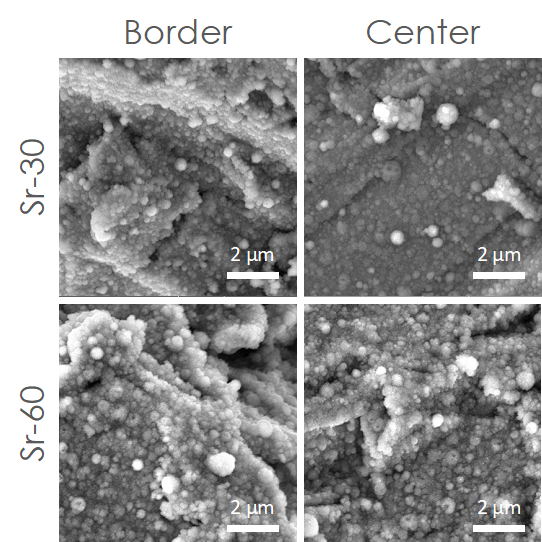

Supplement: Supplementary file 5 [file Image2.TIF]

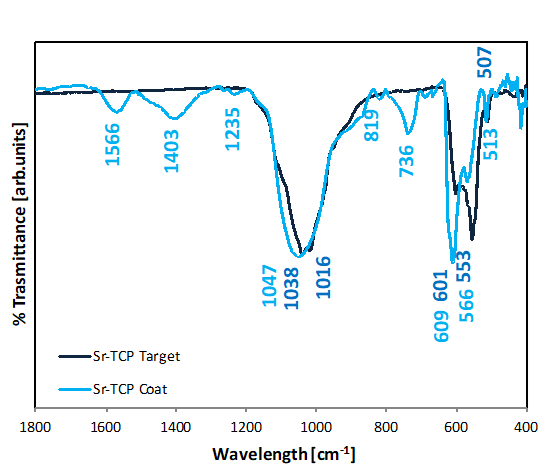

Supplement: Supplementary file 8 [file Image5.TIF]

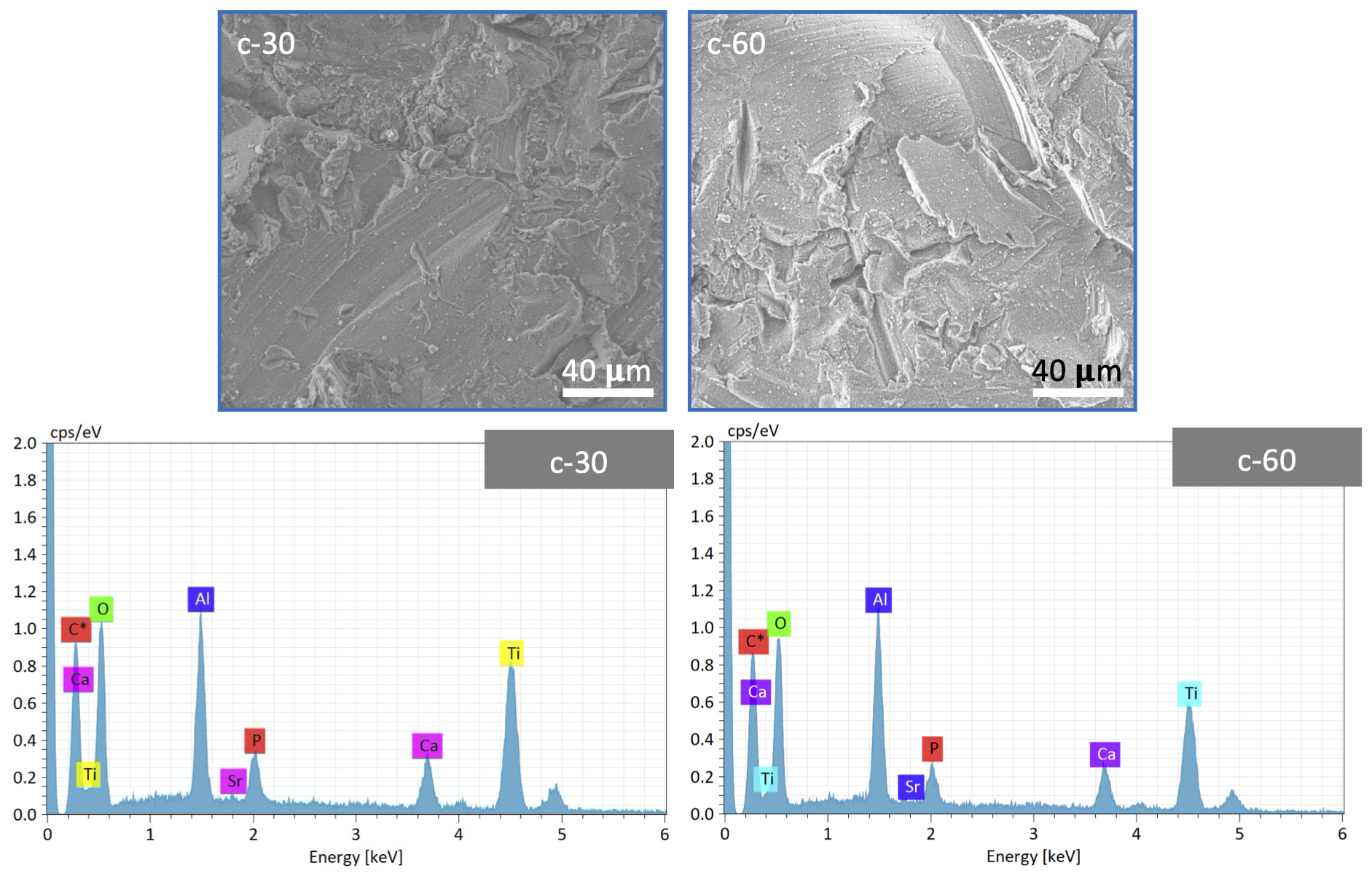

Supplement: Supplementary file 9 [file Image4.TIFF]
