# Supplementary material for: New strontium-based coatings show activity against pathogenic bacteria in spine infection
Source: Front Bioeng Biotechnol. 2024 Apr 10;12:1347811. doi: 10.3389/fbioe.2024.1347811 (PMC11044685; doi:10.3389/fbioe.2024.1347811)
Supplement: Supplementary file 3 [file Table1.docx]

**Supplementary Table S1**. The solubility of Sr-TCP ceramic in the saline solution.

|  | **Element amount (mg/L)** | |
| --- | --- | --- |
| **Time (days)** | **Ca** | **Sr** |
| 1 | 7.81 | 0.30 |
| 3 | 9.20 | 0.50 |
| 30 | 7.92 | 0.84 |
