# Supplementary material for: New strontium-based coatings show activity against pathogenic bacteria in spine infection
Source: Front Bioeng Biotechnol. 2024 Apr 10;12:1347811. doi: 10.3389/fbioe.2024.1347811 (PMC11044685; doi:10.3389/fbioe.2024.1347811)
Supplement: Supplementary file 6 [file Table2.docx]

**Supplementary Table S2**. Antibacterial effect of Sr-TCP coated alloys against bacterial planktonic growth.

| **Strain** | **Time** | **Sample** | **Log reduction** | **% CFUs reduction^a^** | **P-value^b^** |
| --- | --- | --- | --- | --- | --- |
| *E. coli* | 4 h | c-30 | 0.07 | 14.89 | 0.4283  (ns) |
|  |  | c-60 | 0.11 | 22.38 | 0.2717  (ns) |
|  | 8 h | c-30 | 0.24 | 42.46 | 0.0007  (***) |
|  |  | c-60 | 0.41 | 61.10 | <0.0001  (****) |
| *S. aureus* | 4 h | c-30 | 0.74 | 81.80 | <0.0001  (****) |
|  |  | c-60 | 0.82 | 84.86 | <0.0001  (****) |
|  | 8 h | c-30 | 0.37 | 57.34 | 0.0055  (**) |
|  |  | c-60 | 0.48 | 66.89 | 0.0005  (***) |

^a^ The percentage of CFUs reduction refers to the total number of viable CFUs per mL in the wells with Sr-TCP coating compared to those with TCP coating.

^b^ Significant p-values are indicated with asterisks: ns = p > 0.05; * = p < 0.05, ** = p < 0.01; *** = p < 0.001; **** = p < 0.0001.
