# Supplementary material for: New strontium-based coatings show activity against pathogenic bacteria in spine infection
Source: Front Bioeng Biotechnol. 2024 Apr 10;12:1347811. doi: 10.3389/fbioe.2024.1347811 (PMC11044685; doi:10.3389/fbioe.2024.1347811)
Supplement: Supplementary file 7 [file Table3.docx]

**Supplementary Table S3**. Anti-adhesion effect of Sr-TCP coated alloys.

|  |  | **Viable bacterial cells** | | | **Biomass staining** | |
| --- | --- | --- | --- | --- | --- | --- |
| **Strain** | **Sample** | **Log reduction** | **% CFUs reduction^a^** | **P-value^b^** | **% reduced biomass^c^** | **P-value^b^** |
| *E. coli* | c-30 | 0.76 | 82.62 | 0.0003  (***) | 15.18 | 0.0284  (*) |
|  | c-60 | 0.52 | 69.80 | 0.0041  (**) | 25.72 | 0.0024  (**) |
| *S. aureus* | c-30 | 0.28 | 47.52 | 0.0891  (ns) | -22.77 | 0.0568  (ns) |
|  | c-60 | 1.09 | 91.87 | <0.0001  (****) | 21.76 | 0.0442  (*) |

^a^ The percentage of CFUs reduction refers to the total number of viable CFUs per alloy adhered on Sr-TCP coating compared to those adhered on TCP coating.

^b^ Significant p-values are indicated with asterisks: ns = p > 0.05; * = p < 0.05, ** = p < 0.01; *** = p < 0.001; **** = p < 0.0001.

^c^ The percentage of reduced biomass refers to the ratio between the OD_595_ values of the CV-stained adherent biomass on the Sr-TCP coating against the TCP coating.
